# Method for Generating Real-Time Indoor Detailed Illuminance Maps Based on Deep Learning with a Single Sensor

**DOI:** 10.3390/s25165154

**Published:** 2025-08-19

**Authors:** Seung-Taek Oh, You-Bin Lee, Jae-Hyun Lim

**Affiliations:** 1 Smart Natural Space Research Center, Kongju National University, Cheonan 31080, Republic of Korea; ost73@kongju.ac.kr; 2 Department of Computer Science & Engineering, Kongju National University, Cheonan 31080, Republic of Korea; youbbqlsl@smail.kongju.ac.kr

**Keywords:** illuminance map, deep learning, single sensor, indoor detailed illuminance

## Abstract

Emerging lighting technology aims to enhance indoor light quality while conserving energy through control systems that integrate with natural light. In related technologies, it is crucial to identify quickly and accurately indoor light environments that are constantly changing due to natural light. Consequently, a large number of sensors must be installed, but installing multiple sensors would cause an increasing data processing load and inconvenience to users’ activities. Some have attempted to calculate natural light characteristics, such as solar radiation and color temperature cycles, and implement natural light lighting technology by applying deep learning technology. However, there are only a few cases of using deep learning to analyze indoor illuminance, which is essential for commercializing natural light lighting technology. Research on minimizing the number of sensors is also lacking. This paper proposes a method for generating a detailed indoor illuminance map using deep learning, which calculates the illuminance values of the entire indoor area with a single illuminance sensor. A dataset was constructed by collecting dynamically changing indoor illuminance and the position of the sun, and a single sensor was selected through analysis. Then, a DNN model was built to calculate the illuminance of every region of an indoor space by inputting the illuminance measured by a single sensor and the position of the sun, and it was applied to generate a detailed indoor illuminance map. Research has demonstrated that calculating the illuminance levels across an entire indoor area is feasible. Specifically, on clear days with a color temperature anomaly of about 1%, a detailed illuminance map of the indoor space was created, achieving an average MAE of 2.0 Lux or an MAPE of 2.5%.

## 1. Introduction

Recently, system lighting technology that utilizes incoming natural light to provide appropriate illumination levels for each indoor area has gained importance [1,2,3]. Natural light constantly changes depending on the sun’s position, weather, and climate, and thus has different effects on each indoor area [4,5]. Incoming natural light can create an uneven lighting environment in the room, depending on the time and location. However, controlling real-time lighting systems to solve this problem is complex [5,6]. To precisely control system lighting, it is essential to accurately measure illuminance at multiple points in the room in real time. The most common method is to install various sensors throughout the indoor area to measure the illuminance at each point [7,8]. Various illumination distributions in the natural light inflow environment can be accurately identified, and the system lighting technology linked to this can be realized only when many sensors are installed [9]. However, when multiple sensors are installed, this can cause space constraints and inconvenience to users’ activities [10], or problems such as network overload, energy consumption, data delay, and system failure in collecting sensing data may occur [9,11]. Furthermore, a large amount of sensing data requires considerable cost and time for processing and analysis. These problems make it challenging to implement a system lighting technology that monitors indoor lighting status in real time or responds to dynamic changes in natural light [12]. To commercialize a lighting technology using a natural light-linked system, there is a need to reduce or eliminate the reliance on indoor sensors; however, related research is lacking.

Deep learning is a field of machine learning that recognizes spatiotemporal patterns and information through learning from a large amount of accumulated datasets [13]. With the development of big data and IoT technology, it has become easier to collect high-quality data, and deep learning technology has been applied in various industries and research fields [14]. Research cases that use deep learning technology are also being introduced in the lighting technology field. Deep learning technology has been applied to recognize the relative positions of people and lighting automatically, and to control lighting or develop a distributed intelligent lighting system [15,16]. Additionally, deep learning technology was recommended to replace complex daylight simulations in predicting sunlight inside a building [13]. An artificial neural network (ANN) was proposed to design an innovative lighting system for personalized lighting control and sunlight harvesting [17]. A GAN model was applied in a study on real-time/non-real-time synchronization control of indoor lighting, which is linked to the color temperature characteristics of natural light [18]. L-Thanh et al. presented a machine learning (ML)-based framework for verifying real-time daylight performance through a simulation method that excludes physical sensors and derives and applies building characteristics [19]. Li et al. introduced a daylight prediction system based on a GAN model that takes as input all building elements, including images and numerical data, with the objectives of improving the accuracy and generalization of natural light-related information [20]. In previous cases, calculating indoor illuminance required complex and diverse parameters. There have been approaches in the architectural field that integrated deep learning with simulation-based data. However, there have been few research studies on analyzing actual illuminance or creating detailed indoor illuminance maps by using deep learning to reduce the number of sensors needed.

This study proposes a technology for generating an illuminance map that calculates detailed and accurate indoor lighting using a single sensor. A dataset of indoor illuminance by distance from the window side, based on the incoming natural light, was constructed and analyzed. Then, a DNN model was designed and built to measure illuminance through a single sensor installed on the window side. The measured values were input with the sun’s position information to calculate detailed illuminance indoors. Following this, the model’s performance was optimized through parameter tuning, while a real-time illuminance map was calculated and provided. This process provided a method to minimize the illuminance sensors required for natural light-linked system lighting technology.

## 2. A Method for Generating Real-Time Indoor Illuminance Maps with a Single Sensor

**Figure 1:**
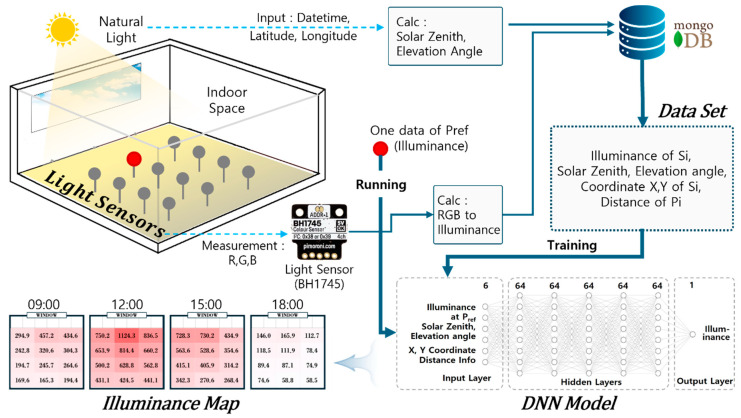
Overview of the proposed method.

This study proposes a method to derive a detailed illuminance distribution of an entire room using illuminance values acquired through a single sensor. To achieve this, an experimental environment was first constructed. The hourly illuminance changes at multiple points indoors were then measured, and the sun’s position information, as well as the sensor’s position and distance, was derived. These data were stored in a database. Then, a dataset for learning was constructed based on this information. After that, a deep learning model for generating an illuminance map was designed, and dataset-based learning was performed to create an illuminance map of the entire indoor area using only input data from a single point indoors. Figure 1 is an overview of the proposed method.

### 2.1. Building an Experimental Environment and Learning Dataset

**Figure 2:**
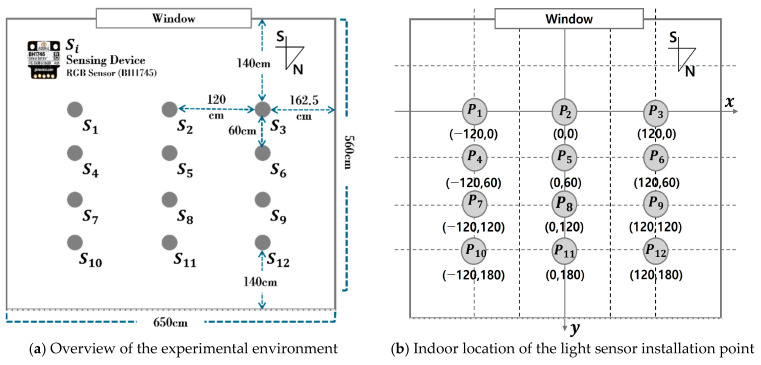
Experimental environment.

Training data is required to develop a deep learning model that generates a detailed illuminance map using the illuminance of a single point acquired through a single sensor installed indoors. Therefore, an experimental environment was built to implement the proposed method. A laboratory at K University, situated at 36.85° latitude and 127.15° longitude, was selected as the experimental space. The experimental site faces south and is equipped with illuminance-measuring equipment to estimate illuminance, taking into account the influence of natural light. Figure 2 provides an overview of the experimental environment configuration, with the distance and positional relationships for each point also indicated.

The experimental space in Figure 2 measures 650 × 560 × 264 cm (W × D × H) in size and features one window, which is 650 × 300 cm (W × H) in size. The room was divided into equal parts, and RGB sensor-based illuminance sensing devices were fabricated and installed at 12 points as in (a). The sensing device utilized an RGB sensor (BH1745, Pimoroni, Sheffield, UK) that measures the R, G, and B color values of light and then calculates illuminance based on these values. The sensing device was installed on an embedded development board (ESP32-S3-DevKitC-1, Espressif, Shanghai, China), equipped with a Wi-Fi module, to enable wireless transmission and reception of the sensed values. To measure stable optical characteristics, an optical diffuser was used to evenly disperse the light incident on the sensor [21]. Additionally, the Auto Integration function was applied to prevent saturation that can occur with high light intensity. This setup helped avoid abnormal indoor illuminance measurements. In cases where the wireless transmission and reception functions of certain sensors were limited, the corresponding values were excluded from the dataset composition. Afterwards, calibration was performed using the reference equipment (spectroradiometer CAS-140CT, Instruments, Munich, Germany) in a light-blocked environment. According to Korean measurement standards, it was installed 80 cm from the floor. (b) shows the relative position of each sensor installation point ($P_{i}$) in X, Y coordinates with the center point $P_{2}$ on the window side as the origin (0, 0). Through the experimental environment depicted in Figure 2, the indoor illuminance values for each point were collected every minute, from sunrise to sunset, for approximately 175 days, from 29 March 2024 to 1 September 2024, and stored in a MongoDB-based database. The illuminance was calculated using the library provided by the sensor manufacturer, based on the R, G, and B channel values collected at each point through MongoDB [22]. Table 1 presents an example of the indoor illuminance dataset configured for learning the proposed method.

As shown in Table 1, the indoor illuminance dataset included the sun’s position information, which affected the direction and intensity of natural light, as well as the measurement time and illuminance at each indoor sensor installation location. The azimuth and elevation angles, which provide positional information about the sun, were calculated using the date, time, latitude, and longitude information through a function provided by the National Oceanic and Atmospheric Administration (NOAA) [23,24]. The illuminance at 12 indoor points that changed over time was measured, calculated, and stored every minute using a sensing device. Since the illuminance of the experimental space exhibited a constant distribution and a change pattern of 0 to approximately 1400 Lux, it was accumulated and collected without separate preprocessing, such as scaling.

### 2.2. Correlation Analysis and Selection of Optimal Input Factors

**Figure 3:**
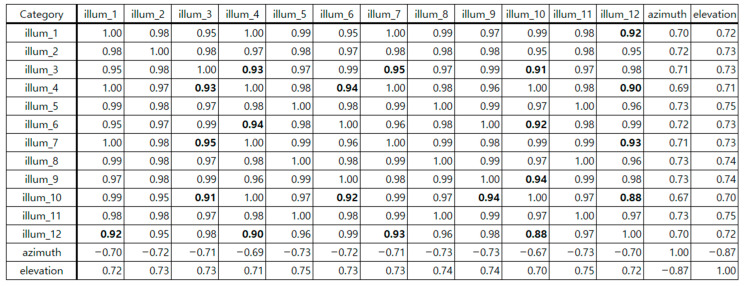
Correlation analysis (illuminance values for each sensor location and sun position information).

After constructing the dataset, the optimal position of a single sensor was set. The collected data was analyzed to determine the optimal location of a single sensor and the input factors of the deep learning model for generating a detailed indoor illuminance map. The correlation between azimuth and elevation and the illuminance at 12 indoor locations was analyzed, and the results are shown in Figure 3.

**Figure 4:**
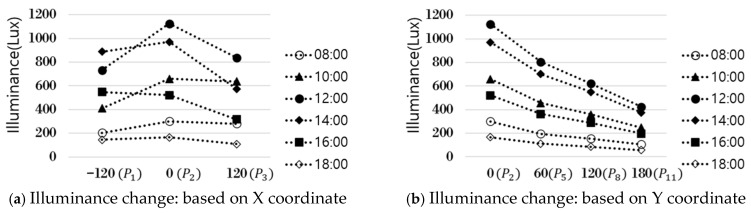
Analysis of illuminance changes by distance.

As shown in Figure 3, the correlation coefficient by illuminance at all points was relatively high, ranging from 0.88 to 1.00. The illuminance between each point was interrelated and worked organically to create the overall lighting environment of the room. Points with a correlation of less than 0.95 were identified and marked in bold letters to find points with high correlation. The illuminance measured at points $P_{2}$, $P_{5}$, $P_{8}$, and $P_{11}$, which correspond to the center of the room (on the X-axis), showed a high correlation (over 0.95) with the illuminance of all other points. At the remaining points, one to four values of less than 0.95 were identified. The change in illuminance according to the X and Y coordinates was analyzed to confirm the change in illuminance at each indoor point over time. The effect of natural light on indoor illuminance over time on a clear day (7 April 2024) was examined, with the percentage of CCT differences of 5% or more per minute being approximately 1% [21]. On the X-axis, the illuminance changes at points $P_{1}$, $P_{2}$, and $P_{3}$ were analyzed. Meanwhile, the illuminance changes at points $P_{2}$, $P_{5}$, $P_{8}$, and $P_{11}$ on the Y-axis were analyzed, and the results are presented in Figure 4.

As shown in Figure 4a, immediately after sunrise (8:00) and just before sunset (18:00), the amount of natural light was negligible. Hence, the illuminance value was low, and the difference in the X-axis position was slight. However, at noon, when the sun was at its highest altitude, the illuminance increased rapidly from 732 Lux to 1124 Lux depending on the X coordinate and then decreased to 836 Lux. The difference in illuminance between the center and the two ends was the largest. This phenomenon occurred when the amount of natural light from the center point increased rapidly at that time, resulting in a substantial increase in illuminance. At 10 o’clock, when the sun was in the eastern sky, the illuminance increased toward point P_3_. Conversely, at 16 o’clock, when the sun was in the western sky, the illuminance increased toward point P1. The analysis results of illuminance changes according to the Y coordinate of points P_2_, P_5_, P_8_, and P_11_ are shown in Figure 4b. Overall, the illuminance was high at P_2_ at the window side, and the illuminance decreased as the Y coordinate increased, or when it moved away from the window. The range of illuminance changes was different depending on the time zone. At 8 o’clock and 10 o’clock, when natural light was less, the difference in illuminance according to the Y coordinate was not significant, and the overall low illuminance was maintained consistently without significant fluctuations at below 200 Lux. On the other hand, in the remaining time zones where the amount of natural light was substantial, the change in illuminance by hour was also significant, and the difference in illuminance between the window side and the interior of the room was quite distinct. Based on these analysis results, the location of a single sensor was determined. The change in illuminance by hour at points in the left and right directions of P_2_, P_5_, P_8_, and P_11_, which were the central axis points along the X-axis of the indoor space, changed in various patterns. Considering the Y-axis direction, the change in illuminance was the greatest at P_2_; therefore, P_2_ was selected as the most advantageous point to identify the influence of natural light that constantly changes and flows in. The P_2_ point was set as the installation location (P_ref_) of a single sensor, and the illuminance at that point was selected as a factor of the deep learning model for generating an indoor illuminance map. Based on the illuminance of P_ref_ and the distance between the predicted point P_n_ and P_ref_ along the X and Y axes, it would be possible to predict the illuminance of P_n_ and generate a detailed map of indoor illuminance. Through an analysis of the illuminance changes by time zone, the azimuth and elevation angles, which are the positional information of the sun that change over time, are also crucial factors in determining the amount of natural light entering the room. Accordingly, the input parameters of the proposed model were set as the illuminance of P_ref_, the coordinates of the predicted point, the distance between P_ref_ and the predicted point, the azimuth, and elevation angles of the sun.

### 2.3. DNN Model for Generating Indoor Illuminance Maps

**Figure 5:**
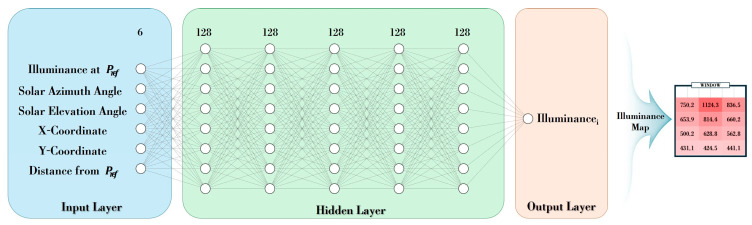
Deep learning model for generating detailed indoor illuminance maps.

A deep learning model that generates an indoor illuminance map was developed using selected input factors, including the illuminance value of a single sensor. The deep learning model was implemented using the Pytorch (2.4.1) framework in a Python 3.11-based Anaconda environment. For learning and execution, a PC with Windows 10 OS, AMD Ryzen 5 5600X CPU (AMD, Santa Clara, CA, USA), GeForce GTX 1650 (NVIDIA, Santa Clara, CA, USA), and 16 GB memory was adopted. In this hardware environment, a deep learning model was designed to generate an indoor, detailed illuminance map that learns the relationship between the illuminance acquired through a single sensor, the sun’s elevation angle and azimuth angle, and the indoor multiple-view illuminance. Figure 5 shows the resulting design of the proposed model.

As shown in Figure 5, the model’s input parameters are the illuminance at the point $P_{ref}$, the azimuth of the sun, the elevation of the sun, the X, Y coordinates of the predicted point $P_{n}$ (n ≠ 2), and the distance between points $P_{n}$ and $P_{ref}$. The hidden layers of the initial DNN model consisted of five fully connected layers, each with 128 nodes. ReLU was used as the activation function in each of the hidden layers. Adam was used as the model’s optimizer, and the mean absolute error (MAE) was used as the loss function. The hidden layer learned the positional information of the sun and the relationship between two points. The output layer calculated the illuminance value (Illuminance_i_) at the corresponding predicted point (P_n_). After that, the illuminance for each predicted point was comprehensively reconstructed to generate an illuminance map consisting of rows and columns of a specific size. Learning and validation of the model were conducted by splitting the dataset in an 80:20 ratio, with the days selected for later performance evaluation excluded from the dataset. After confirming that the proposed model can calculate illuminance at each point in Figure 5, the deep learning model was optimized to derive more accurate results. Hyperparameter tuning was performed to improve the performance of the deep learning model that calculates illuminance for the entire indoor area [25,26]. In the tuning process, the grid search technique was utilized to verify various combinations of batch sizes, learning rates, and the number of nodes in the hidden layer. The hyperparameter setting variables for tuning are as shown in Table 2.

**Figure 6:**
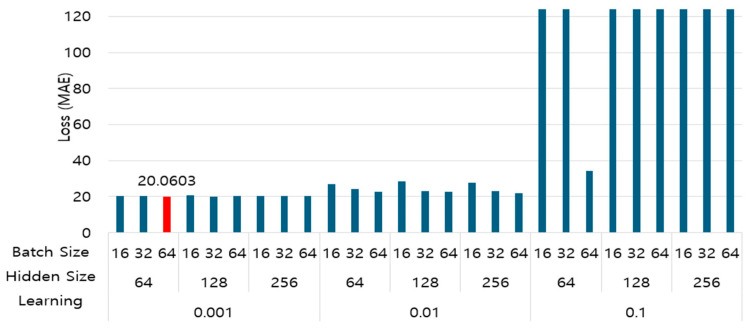
Hyperparameter tuning results.

As shown in Table 2, after dividing Batch Size, Hidden Size, and Learning Rate into three stages each, the operating performance of 27 models that combined the setting variables of each parameter was compared and analyzed. The model was trained with each hyperparameter combination, and the performance was evaluated. At this time, the training and verification data were split at an 80:20 ratio. The number of model training iterations for each combination was set to 10. Adam was the optimizer, and the mean absolute error (MAE) was applied to the loss function. The performance evaluation results for each model are shown in Figure 6.

As shown in the results in Figure 6, the loss value was significant when the learning rate was 0.1. It was mainly low and stable when the learning rate was 0.001. Furthermore, performance differences occurred depending on the batch size and the number of nodes in the hidden layer. However, when the learning rate was 0.001, the performance differences in terms of batch size and hidden layer size were not significant. The best-performing model was a combination of a batch size of 64, several nodes in the hidden layer of 64, and a learning rate of 0.001, which resulted in the lowest loss rate of 20.0603. This combination was finally selected and applied to the proposed model. 

**Figure 7:**
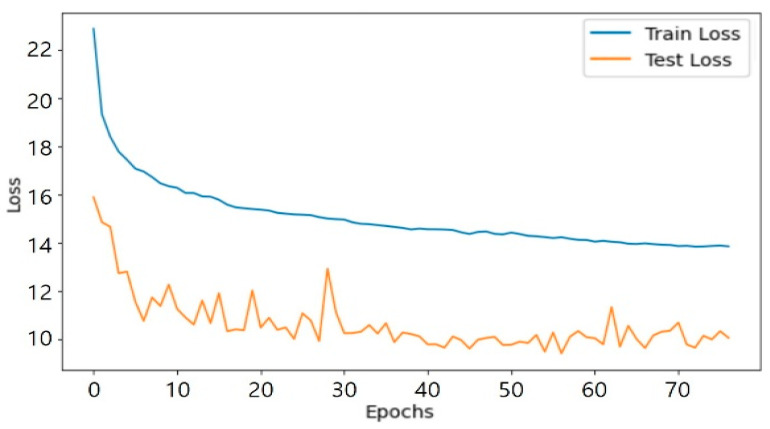
Train loss curve.

As shown in Figure 7, the number of epochs for repetitive learning was set to a maximum of 100, and the early termination condition was set to more than 20 times. The loss value of each execution result was compared. The epoch 77 condition, which showed a Train Loss of 13.8584 and a Test Loss of 10.0632, was applied. The proposed model calculated the illuminance at a specific point. At this time, it was also possible to calculate the illuminance at an arbitrary location (distance) other than P_i_ set in the pre-experimental environment. Afterwards, the illuminance values of each point were integrated and configured to generate a detailed indoor illuminance map that visually expressed the illuminance distribution in each indoor area.

## 3. Experimental Results and Discussion

**Figure 8:**
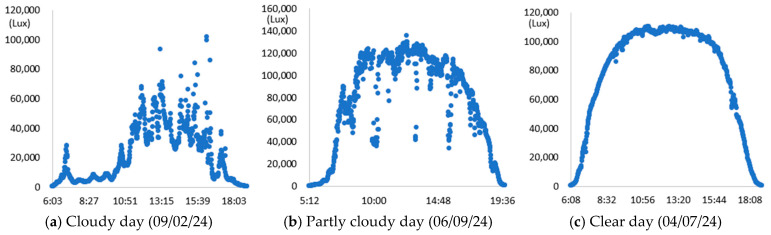
Changes in illuminance by weather (daily).

To evaluate the performance of the proposed model, experimental days were selected considering various weather conditions. All selected days were excluded from the learning and validation datasets of deep learning. These three days were a clear day (7 April 2024, with an outlier rate of 1.26%), a partly cloudy day (9 June 2024, with an outlier rate of 18.21%), and an overcast day (2 September 2024, with an outlier rate of 18.14%). A case where the color temperature difference at every 1 min interval was 5% or more was considered an outlier. A previous study that distinguished the degree of brightness and cloudiness based on the outlier rate was cited [21]. The illuminance measurements collected at an outdoor location to confirm the daily change in natural light for each selected day are presented in Figure 8.

In Figure 8, on a cloudy day (a), the measured illuminance at 1 min intervals was pretty uneven and showed a substantial deviation. On some cloudy days (b), sections where the illuminance values fluctuated were observed in the middle of the dataset. In contrast, the illuminance displayed a consistent parabolic pattern on clear days (c). As illustrated in Figure 8, the proposed method was applied to each selected day, where variations in natural light depended significantly on the weather, in order to create hourly illuminance maps. Sensor S_2_, located at point P_2_, was designated as the primary illuminance sensor, and the illuminance values for the remaining points (P_1_, P_3_–P_12_) were calculated based on the readings from this sensor. Considering sunrise and sunset in Korea, as well as business hours, detailed indoor illuminance maps for four time zones—9:00, 12:00, 15:00, and 18:00—were generated. The results of the measured illuminance and the detailed illuminance map are compared in Table 3.

As shown in Table 3, we compared the application results of the proposed method with measurements over time, also analyzing the associated error and error rate. In Table 3, cases where the error rate for illuminance at each point exceeded 5% are highlighted in bold. On cloudy days, specifically 2 September 2024, the average MAPE was approximately 15%, indicating a high error rate. Figure 8a illustrates that when natural light illuminance was inconsistent or fluctuated rapidly, it complicated the generation of an accurate illuminance map. Conversely, on certain cloudy days, such as 7 June 2024, where there were occasional rapid variations in illuminance throughout the day, we achieved more favorable results. During this time, we generated a highly accurate indoor illuminance map with an MAPE of 4.07% or less. However, this outcome was influenced by hourly aggregation, and we anticipated that accuracy would decline in sections with rapid changes in illuminance. However, the results for a clear day, specifically 7 April 2024, demonstrated highly accurate illuminance measurements, with an MAPE ranging from 1.17% to 2.78%. Notably, at 12:00 and 15:00, when natural light was at its peak, the MAPE was exceptionally low, at 1.59% or less. This indicates that the proposed method performed remarkably well under clear weather conditions, where the availability of natural light is high. Table 4 presents the experimental results obtained on clear days.

Table 4a shows the measurement results using the sensor, and Table 4b shows the results obtained by applying the proposed method. The results for all time zones showed that the illuminance gradually decreased as the distance from the window increased. At 09:00, the right side of the room had higher illuminance than the left side, and the opposite trend was observed at 15:00. At 12:00, the overall illuminance was the highest. At 18:00, around sunset, the overall illuminance was low. In general, it demonstrated robust illuminance calculation performance at P_1_, which is the location closest to the installation site of a single illuminance sensor, and at P_12_, which is the farthest. Table 4 shows that the proposed model effectively learned and calculated the changes in illuminance at each location based on the sun’s position on a clear day. The maximum illuminance error was approximately 18 lux, resulting in a maximum error rate of 2.78%. This indicates that the model produced an accurate illuminance map. Overall, the findings confirm that the proposed model can perform exceptionally well in clear weather conditions, which is expected to be advantageous for future energy savings.

This study presents an empirical method for generating detailed illuminance maps for various spaces using only a single sensor, provided that pre-learning is conducted with measured data. However, the performance of this method can vary depending on natural light patterns under different weather conditions. While improvements are anticipated when training utilizes a comprehensive dataset that includes all seasons and times, creating accurate illuminance maps may still be challenging when natural light changes rapidly. To overcome this limitation, further research is necessary to incorporate additional parameters, such as indoor images, and to establish a connection with lighting systems. In the commercialization phase, we plan to develop an efficient control strategy and quantitatively evaluate the effects of lighting and wireless network operation.

## 4. Conclusions

Recently, natural light-linked lighting systems that control indoor lighting environments in response to incoming natural light have been highlighted. However, there is a limitation that a considerable number of sensors must be installed indoors, making it challenging to generalize related technologies. This paper proposes a method for generating a deep learning-based indoor detailed illuminance map that calculates the illuminance of an indoor area using illuminance data acquired through a single sensor. An experimental environment was set up first, by installing optical characteristic sensors at 12 points that evenly divided the indoor space. An indoor illuminance dataset spanning approximately 6 months was acquired through an experimental environment. Afterwards, through analysis, the location (Pref) of a single sensor, which was used to identify indoor illuminance changes caused by incoming natural light, was selected as the center point on the window side. A deep learning model was designed to calculate the illuminance of each indoor area, considering the distance from a single sensor location. The illuminance of a single sensor location, the positional information of the sun’s elevation and azimuth angles, and the coordinates and distance information of the predicted point were input into the deep learning model. The proposed model was optimized and built by setting the Batch Size, Hidden Size, and Learning Rate to 64, 64, and 0.01, respectively, through hyperparameter tuning. The illuminance of a specific indoor point was calculated through the deep learning model, and a 3 × 4 detailed indoor illuminance map was generated by synthesizing the calculated illuminance values, allowing the user to create an illuminance map with the desired specifications. To evaluate performance, we selected one clear day, one partly cloudy day, and one cloudy day, each with a different percentage of color temperature outliers. The proposed method was then applied to generate illuminance maps. The results demonstrated that the performance of illuminance map generation was significantly better on clear and partly cloudy days compared to cloudy days, achieving an MAPE of approximately 4%. Notably, on clear days, the illuminance in all indoor areas was calculated with a high level of accuracy, resulting in an average MAPE error rate of 2.00% for each time zone.

In the future, research is planned to improve the method of generating detailed indoor illuminance maps by securing additional indoor datasets for an extended period after configuring more diverse experimental environments. A follow-up study will also be conducted to generalize the application of natural lighting systems by enabling them to respond to various weather and climate conditions. Efforts will continue to develop a sensor-less natural lighting system that links indoor and sky images, as well as satellite images, to eliminate the need for sensors completely.

## Figures and Tables

**Table 1:** Indoor illuminance dataset.

Time	Solar Information		Illuminance (Lux)								
	Azimuth	Elevation	$P_{1}$	$P_{2}$	–	$P_{7}$	$P_{8}$	$P_{9}$	$P_{10}$	$P_{11}$	$P_{12}$
03/29/24 06:32	86.62	1.58	15	20	–	16	28	22	7	6	7
–	–	–	–	–	–	–	–	–	–	–	–
03/29/24 09:52	121.58	39.75	379	614	–	392	471	646	338	329	400
03/29/24 09:53	121.82	39.92	388	635	–	390	470	634	341	327	399
–	–	–	–	–	–	–	–	–	–	–	–
09/01/24 12:30	295.24	−17.98	504	766	–	440	512	548	369	319	331

**Table 2:** Set parameters for hyperparameter tuning.

Hyperparameter	Batch Size	Hidden Size	Learning Rate
Value	[16, 32, 64]	[64, 128, 256]	[0.1, 0.01, 0.001]

**Table 3:** Performance evaluation results of the illuminance map production.

Category		Comparison of Calculated Results and Actual Measurements: Illuminance (Lux)											
Time		9:00			12:00			15:00			18:00		
Date	Position	Calculation	Measurement	Error	Calculation	Measurement	Error	Calculation	Measurement	Error	Calculation	Measurement	Error
09/02/24 (cloudy day)	P_1_	63.9	57.6	6.3	553.7	593.6	39.9	575.4	451.6	123.8	71.8	89.5	17.7
	P_3_	59.6	71.5	11.9	595.1	493.9	101.2	363.5	355.3	8.1	61.5	54.1	7.4
	P_4_	50.3	57.7	3.3	485.5	475.7	9.8	465	311.7	153.3	52.6	72.5	19.9
	P_5_	57.7	62.7	5	582.8	565	17.8	431.2	372.1	59	58.6	73	14.4
	–												
	P_12_	31.5	34.6	3.1	317.6	256.6	61	220.3	188.5	31.8	31.1	30.6	0.5
	MAE	4.88 (MAPE: 9.43%)			45.39 (MAPE: 12.56%)			59.95 (MAPE: 21.36%)			10.00 (MAPE: 16.63%)		
06/09/24 (partly cloudy day)	P1	334.5	353.2	18.8	466.5	516.4	49.9	464.4	470.5	6.1	187.3	187.1	0.2
	P3	424.6	434.2	9.6	513.9	515.3	1.3	342.8	348.5	5.7	149.7	150.5	0.8
	P4	285.8	357.1	0.1	393.2	419.2	26	365.7	366.6	0.9	151.7	143.2	8.6
	P5	357.1	306	3.1	479.7	487.2	7.5	377.3	379.4	2.1	149	146	3
	–												
	P12	204.4	205.3	0.8	254.7	271.8	17.1	195.4	188.6	6.8	82.3	81.4	0.9
	MAE	5.07 (MAPE: 2.11%)			15.21 (MAPE: 4.07%)			5.44 (MAPE: 2.11%)			2.65 (MAPE: 2.26%)		
04/07/24 (clear day)	P1	294.9	294.5	0.4	750.2	732.5	17.7	728.3	739.4	11.1	146.0	143.9	2.0
	P3	434.6	447.2	12.6	836.5	837.0	0.5	434.9	426.5	8.4	112.7	109.2	3.6
	P4	242.8	235.1	7.7	653.9	640.4	13.5	563.6	581.2	17.6	118.5	115.2	3.3
	P5	320.6	306.0	14.7	814.4	803.9	10.4	528.6	519.4	9.2	111.9	111.6	0.4
	–												
	P12	194.4	200.8	6.4	441.1	452.5	11.4	268.4	268.1	0.3	58.5	58.7	0.2
	MAE	6.38 (MAPE: 2.69%)			7.27 (MAPE: 1.44%)			6.91 (MAPE: 2.07%)			2.38 (MAPE: 1.89%)		

**Table 4:** The outcome of generating an hourly illuminance map for a clear day on 7 April 2024.

Time	09:00	12:00	15:00	18:00
(**a**) Measurement	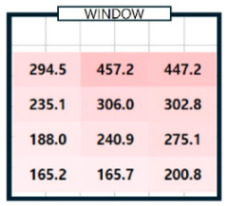	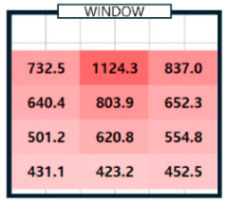	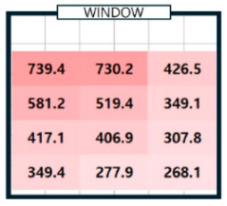	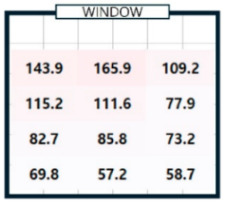
(**b**) Calculation	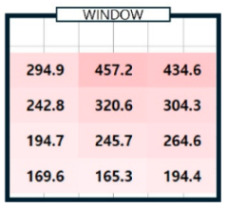	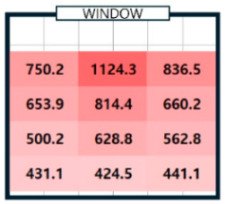	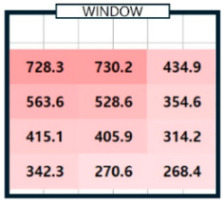	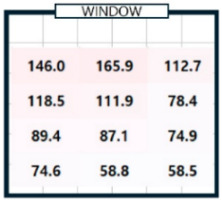

## Data Availability

The data presented in this study are only available on request from the corresponding author due to legal reasons.
